# Cellular and molecular phenotypes of proliferating stromal cells from human carcinomas

**DOI:** 10.1038/sj.bjc.6605652

**Published:** 2010-04-20

**Authors:** E P Kopantzev, N A Vayshlya, M R Kopantseva, V I Egorov, M Pikunov, M V Zinovyeva, T V Vinogradova, I B Zborovskaya, E D Sverdlov

**Affiliations:** 1 M M Shemyakin and Yu A Ovchinnikov Institute of Bioorganic Chemistry, Russian Academy of Sciences, Ul Miklukho-Maklaya 16/10, 117997, Moscow, Russia; 2 A V Vishnevsky Institute of Surgery, Ul B Serpuchovskaya 27, 115998, Moscow, Russia; 3 Engelhardt Institute of Molecular Biology, Russian Academy of Sciences, Ul Vavilova 32, 119991, Moscow, Russia; 4 Institute of Carcinogenesis, Cancer Research Center of Russian Academy of Medical Sciences, Kashirskoye sh., 24, 115478, Moscow, Russia

**Keywords:** microenvironment, stromal cells, fibroblast, lung, carcinoma

## Abstract

**Background:**

Stromal cells are a functionally important component of human carcinomas. The aim of this study was to obtain and characterise primary cultures of stromal cells from human carcinomas and the corresponding surrounding normal tissue.

**Methods:**

Primary stromal cell cultures from tumours of lung, oesophagus and pancreas were obtained using a mild tissue dissociation method and a medium for culturing mesenchymal cells. Immunofluorescence staining and western blotting were used to analyse the expression of differentiation markers and selected known oncoproteins in the cell cultures obtained.

**Results:**

A panel of stromal primary cultures was prepared from different human tumours and from matched normal cancer-free tissues. The *in vitro* proliferative potential of tumour-associated fibroblasts was shown to be higher than that of matched normal stromal cells. A mutational analysis of the *TP53* and *KRAS2* genes in a number of stromal cultures did not reveal known mutations in most cells of the cultures studied. Western blot analysis showed that stromal cells of lung tumours were characterised by a statistically significantly lower expression level of the p16 protein as compared with that in normal lung stromal cells. An important finding of our study was that, according to immunofluorescence assay, a fraction of fibroblast-like vimentin-positive cells in some tumour and normal stromal cell cultures expressed an epithelial marker – cytokeratins.

**Conclusions:**

Proliferating stromal cells from the carcinomas studied proved to be genetically normal cells with altered expression profiles of some genes involved in carcinogenesis, as compared with normal stromal cells. Epithelial-mesenchymal transition may lead to the emergence of transdifferentiated fibroblast-like cells in tumour stroma and in the tumour-surrounding tissue.

Virtually all carcinomas evolve as complex mixtures of genetically altered epithelial cells and presumably genetically normal host stromal cells such as fibroblasts, smooth muscle cells, vascular and inflammatory cells (Mueller and Fusenig, 2004). The spatial and temporal complexity of cancer stroma is now well recognised. It has been shown that stromal alterations can precede malignant progression of epithelial tumours (Bhowmick *et al*, 2004). The alterations can include emergence of activated stromal fibroblasts (myofibroblasts) in the connective tissue surrounding cancer cells in a majority of epithelial tumours. Several lines of studies performed *in vitro* and *in vivo* indicated that the proliferative and invasive potential of malignant epithelial cells was modulated through heterotypic interactions with the mesenchymally derived stromal microenvironment (Elenbaas and Weinberg, 2001; Tlsty and Hein, 2001; Kalluri and Zeisberg, 2006). Microenvironmental conditions within the tumour, induced by activated stromal cells, such as disorganised vascular network, increased interstitial fluid pressure and intratumoural hypoxia, which hindered the efficient delivery and action of anticancer drugs. Although tumour stromal cells might be an attractive target for anticancer therapy, molecular differentiation of tumour-associated fibroblasts and their activated signaling pathways are still poorly studied.

One of the most powerful tools for deciphering molecular mechanisms of cancer progression and understanding of tumour intervention strategies are cell lines derived from human cancer cells. It was shown that established human cancer cell lines retained morphological, phenotypic and genetic characteristics of the corresponding parental tumours (Wistuba *et al*, 1998, 1999). We supposed that stromal cell cultures established from human tumours might represent the corresponding tumour microenvironment, thus providing a useful complement to cancer cell lines in human neoplasia research.

In this study, we describe a preparation of a panel of stromal cell cultures derived from several types of human tumours and from morphologically normal, cancer-free tissue. We found that common features of all tumour stromal cells were extended replicative lifespan in culture and a higher Ki67 labeling index compared with matched normal stromal cells. A mutational analysis of the *TP53* and *KRAS2* genes in a number of stromal cultures studied revealed that most cells were genetically normal. An important finding of our studies is that, as shown by immunofluorescence assay, a fraction of fibroblast-like cells in some tumour and normal stromal cell cultures expressed both epithelial and mesenchymal markers, cytokeratins and vimentin, suggesting that these cells have undergone epithelial-mesenchymal transition (EMT). Western blot analysis of differentiation markers and known oncoproteins revealed heterogeneous patterns of their expression that varied among individual cancer stromal cultures, even of the same histological type. Stromal cells of lung tumours were characterised by a statistically significantly lower expression level of the p16 protein as compared with that in normal lung stromal cells. A common feature of all the cultures was a low content of the p53 protein that may be indicative of the wild-type p53 in the cells analysed.

## Materials and Methods

### Materials and cells

Unless otherwise specified, chemicals were obtained from Sigma-Aldrich (St Louis, MO, USA). Sera and cell culture media were obtained from Invitrogen (Invitrogen Corporation, Carlsbad, CA, USA). Primary antibodies were as follows: mouse monoclonal D-8 anti-survivin, mouse monoclonal DO-1 anti-p53, mouse monoclonal DCS-6 anti-cyclin D1, rabbit polyclonal N-20 anti-p16, rabbit polyclonal H-63 anti-N-cadherin, rabbit polyclonal H-108 anti-E-cadherin, rabbit polyclonal V-18 anti-TCF-3 (E2A), mouse monoclonal 0411 anti-GAPDH (Santa Cruz Biotechnology, Santa Cruz, CA, USA), mouse monoclonal V9 anti-vimentin, mouse monoclonal 1A4 anti-*α*-smooth muscle actin, mouse monoclonal C-11 anti-pancytokeratin (Sigma-Aldrich), mouse monoclonal L70G2 anti-SNAI I (Snail) (Cell Signaling, Danvers, MA, USA), mouse monoclonal anti-Bcl-2 (Upstate, Lake Placid, NY, USA), mouse monoclonal anti-Ki67 (clone MIB-1) and rabbit polyclonal Z0622 anti-pancytokeratin from Dako (Dako, Carpinteria, CA, USA). Secondary goat anti-mouse and anti-rabbit IgG antibodies conjugated to HPR were obtained from Santa Cruz Biotechnology. Secondary goat anti-mouse and anti-rabbit IgG antibodies conjugated to Alexa Fluor 488 and Alexa Fluor 555 were obtained from Molecular Probes (Eugene, OR, USA).

NCI-H23, A549 and MIA PaCa-2 human cancer cell lines were obtained from American Type Cell Collection (ATCC, Manassas, VA, USA) and maintained in Dulbecco's modified Eagle's medium/Ham's F12 (DMEM/F12, 1 : 1) containing 10% fetal calf serum, 2 mM L-glutamine, 100 U ml^–1^ penicillin and 100 *μ*g ml^–1^ streptomycin. A normal human lung embryonic fibroblast culture (LEF) was kindly provided by Dr GT Sukhikh (Research Center of Obstetrics, Gynecology, and Perinatology, Russian Academy of Medical Sciences, Moscow, Russia). All cells were cultured at 37 °C in humidified atmosphere of 95% air and 5% CO_2_.

### Patients and tissue specimens

Surgical tumour specimens were obtained from a series of 20 patients with diagnosed cancer who have undergone complete resection of tumours at the NN Blokhin Russian Cancer Research Center (Moscow, Russia) and the AV Vishnevsky Institute of Surgery (Moscow, Russia), between December 2004 and May 2006. The final diagnosis was confirmed by hematoxylin-eosin staining of paraffin blocks after the operation. None of the patients received chemo- or radiotherapy before surgery. All tumour samples were obtained after informed consent of the patients. The project protocol was approved by the Institutional Review Boards at the NN Blokhin Russian Cancer Research Center and the AV Vishnevsky Institute of Surgery. Patient and tumour characteristics are listed in Table 1.

Samples were dissected from central parts of the tumours and from normal tissue adjacent to the resected region at the time of surgery. Histological examination of tumour samples revealed that more than 75% of the cells had been cancer epithelial cells. Normal samples were histologically normal and looked microscopically like carcinoma-free tissue specimens. The fresh tumour and normal tissue samples were immediately transferred into sterile Hank's balanced salt solution (HBSS; Invitrogen) and stored on ice.

### Fibroblast culture preparation

All tissue samples were processed within the first 24 h. Before tissue dissociation, fat and necrotic areas were dissected from surgical materials, and tissue samples were minced in a small volume of cold HBSS into 1 mm^3^ pieces using sterile scissors. Minced tissues were then dissociated with collagenase type A (1 mg ml^–1^; Roche Applied Science, Penzberg, Germany), dispase (1 mg ml^–1^; Invitrogen) and deoxyribonuclease I (100 units ml^–1^; Sigma-Aldrich) at 37 °C with gentle agitation for 3–4 h in HBSS with 1% fetal calf serum. The dissociated cells were then filtered using 100 *μ*m cell strainers (Becton Dickinson Labware, Franklin Lakes, NJ, USA), and the cellular filtrate centrifuged at 250 **g** for 5 min. The cell pellet was resuspended in DMEM/F12 (1 : 1) containing 10% fetal calf serum, 2 mM L-glutamine, 100 U ml^–1^ penicillin and 100 *μ*g ml^–1^ streptomycin, and viable cell counts were determined by the Trypan blue exclusion test in a hemocytometer. In all, 10^5^ viable cells were plated on standard uncoated Corning Costar 25-cm^2^ flasks (Corning Incorporated, Lowell, MA, USA). The culture medium was changed every second day, and the cultures reached confluency in 10–12 days (passage 0). The cells were split 1 : 4 once a week. A majority of the stromal cell cultures were frozen at passage 2. In all experiments, cells between passages 4 and 6 were used.

### Proliferation assay

Stromal cells were seeded at 10^3^ cells per well in 96-well black plates. The number of cells was determined daily over a 5-day period using the Hoechst 33258 assay (Rago *et al*, 1990) with a GENios Pro plate reader (TECAN, Mannedorf, Switzerland) at the excitation wavelength of 360/35 nm and detection wavelength of 460/10 nm.

### Mutational analysis

A QIAamp DNA Purification Kit (Qiagen, Valencia, CA, USA) was used to isolate genomic DNA from stromal cells and control cancer cell samples.

Exons 5 (211-bp fragment), 6 (182-bp fragment) and 7 (139-bp fragment) of the *TP53* gene were PCR amplified from genomic DNA samples (see PCR primers in Supplementary Table 1). Briefly, 100 ng of genomic DNA were used as a PCR template in a 50-*μ*l reaction mixture. PCR amplification was performed as follows: 3 min at 95 °C, 1 min at 64 °C, 1 min at 72 °C, and then 29 cycles for 1 min at 95 °C, 2 min at 64 °C and 1 min at 72 °C. The PCR products were column purified using a QIAquick PCR Purification Kit (Qiagen) and directly sequenced with dye terminator chemistry using an ABI Prism 3100-Avant Genetic Analyser automatic DNA sequencer (Applied Biosystems Inc, Foster City, CA, USA). The same set of primers was used for PCR and the sequencing. As a positive control for mutation of the *TP53* gene, genomic DNA of NCI-H23 (codon 246 mutation) and MIA PaCa-2 (codon 248 mutation) cancer cell lines were used.

Mutations at codon 12 of the *KRAS2* gene were examined by a slightly modified mutant-allele-specific amplification method, as described by Yamada *et al* (1998). Briefly, 100 ng of genomic DNA were used as a PCR template in a 50-*μ*l reaction mixture. PCR amplification with wild-type primers (Supplementary Table 1) was performed as follows: 3 min at 94 °C, 2 min at 62 °C, 1 min at 72 °C, and then 35 cycles for 1 min at 94 °C, 2 min at 62 °C and 1 min at 72 °C. PCR with mutant-specific primers (Supplementary Table 1) was performed using the same protocol except that the annealing temperature was 68 °C. DNA samples were considered positive for mutation of the *KRAS2* gene when their PCR products were identified as a 180-bp band in 1.5% agarose gel electrophoresis. As a positive control for codon 12 mutation of the *KRAS2* gene, genomic DNAs of NCI-H23 (GGT>TGT), A549 (GGT>AGT) and MIA PaCa-2 (GGT>TGT) cancer cell lines were used.

### Western blot analysis

Cell lysates were prepared from subconfluent cultures. To this end, cells were washed twice with cold PBS and lysed for 30 min in NP-40 lysis buffer (1% NP-40, 0.2% sodium deoxycholate, 200 mM NaCl, 100 mM Tris-HCl, pH 7.5, 2 mM EDTA, 1 mM EGTA, 10 mM NaF, 1 mM Na_3_VO_4_, 1 mM AEBSF, 100 *μ*g ml^–1^ aprotinin, 100 *μ*g ml^–1^ leupeptin and 100 *μ*g ml^–1^ antipain) on ice. The protein concentration in the lysates was determined using a Micro BCA protein assay kit (Pierce, Rockford, IL, USA). Equal amounts of cell lysates (20 *μ*g of protein) were boiled in SDS sample buffer consisting of 1% SDS, 2% 2-mercaptoethanol, and 62 mM Tris-HCl, pH 6.8, subjected to SDS electrophoresis in 10–15% polyacrylamide minigels and then electrotransferred to a PVDF Immobilon-P membrane (Millipore, Bedford, MA, USA) using a Bio-Rad Trans-Blot SD cell (Bio-Rad Laboratories, Hercules, CA, USA). After this, the membranes were blocked with 5% skimmed milk in PBS-T (PBS containing 0.1% Tween 20) for 1 h at room temperature, incubated in PBS-T containing 5% skimmed milk and the relevant primary antibody overnight at 4 °C and finally washed three times with PBS-T. Mouse monoclonal anti-GAPDH antibody (0411) was used as a loading control. After washing, the membranes were incubated in PBS-T containing 5% skimmed milk and goat anti-mouse or anti-rabbit antibody HRP conjugates (Santa Cruz, 1 : 10 000) for 1 h at room temperature. The membranes were finally washed with PBS-T, and specific signals were visualised using an Immun-Star HRP Chemiluminescent detection kit (Bio-Rad) and a Bio-Rad VersaDoc MP4000 imager station. Protein expression levels were quantified by western blot densitometry, and individual protein levels were normalised to GAPDH values.

### Immunofluorescence

For immunofluorescence experiments, stromal cells were cultured overnight on two-well chamber slides (Becton Dickinson Labware). The cells were washed twice with cold PBS, fixed with 4% paraformaldehyde in PBS for 30 min at 4 °C, washed once with cold PBS, permeabilised in PBS containing 0.5% Triton X-100 for 10 min at room temperature, and then blocked overnight with 10% normal goat serum (Invitrogen) in PBS at 4 °C. The blocked cells were incubated with primary antibody in PBS containing 10% normal goat serum for 45 min at room temperature. After further washes with 0.1% Tween 20 in PBS, Alexa Fluor 488 goat anti-mouse IgG secondary antibody or Alexa Fluor 555 goat anti-rabbit IgG (Molecular Probes) secondary antibody were applied for 1 h. After final washing, slides were incubated with DAPI (Molecular Probes, 1 *μ*g ml^–1^ in PBS) to stain nuclei, or phalloidin -TRITC (Sigma-Aldrich, 0.5 *μ*g ml^–1^ in PBS) to stain F-actin for 45 min, washed with PBS and finally mounted with Prolong Antifade reagent (Molecular Probes). Slides were viewed the next day with a Nikon TE2000U microscope equipped with epifluorescence optics (Nikon Europe, Amstelveen, The Netherlands). Images were captured with a cooled CCD camera (DS-5Mc, Nikon). All captured images were imported into Photoshop 7.0 (Adobe Systems Inc., San Jose, CA, USA) as BMP files.

### Statistical analysis

Data were analysed and presented as the mean±s.e.m. *P*-values were determined by Student's *t*-test, and *P*<0.05 was considered statistically significant.

## Results

**Figure 1:**
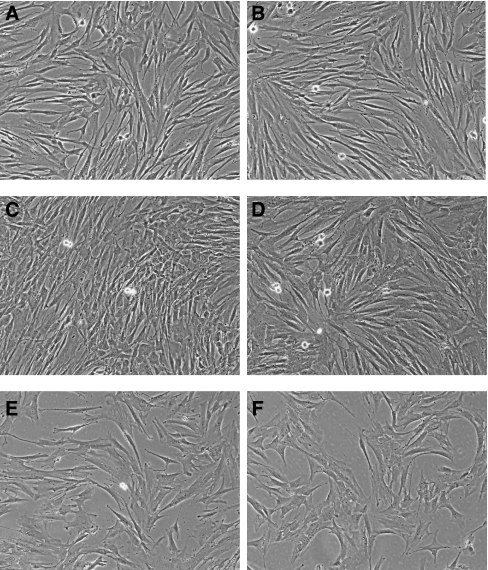
Morphology of normal and tumour stromal cells. Representative microscopic fields showing normal L2N (**A**), L5N (**C**) and tumour L2T (**B**), L5T (**D**), L16T (**E**) P3T (**F**) stromal cells. Original magnification × 100 (phase contrast).

Mild dissociation of surgical specimens and the choice of culture medium for preferential growth of primary mesenchymal cells allowed us to reproducibly obtain morphologically homogenous cultures of stromal cells from a number of human carcinomas, free of contamination with epithelial cells (Figure 1). We have prepared a collection of primary stromal cell cultures from human tumours of various origin and localisation (Table 1). The collection included:

1. A panel of primary cell cultures of lung cancer stromal cells from 15 patients with a confirmed diagnosis of NSCLC (cell cultures L1T-L12T and L14T-L16T). The corresponding stromal cell cultures of unaffected lung tissue from 12 of these patients were also established (cell cultures L1N-L12N). The age of the patients varied from 41 to 69 years (with the mean age of 57 years), and the stages of disease – between IA and IV.
2. Primary stromal cell cultures from primary chondrosarcoma metastasis in lung and from adjacent healthy tissue (cell cultures L13T and L13N, respectively) prepared from one patient.
3. Primary stromal cell cultures (cell cultures E1T and E1N) prepared from one patient with a confirmed diagnosis of oesophagus SCC.
4. Primary tumour stromal cell cultures (cell cultures P2T–P4T) prepared from three patients with a confirmed diagnosis of pancreatic cancer.
5. Primary stromal cell culture P1P prepared from the pancreas of a patient operated on for chronic pancreatitis.

### Cell morphology and proliferative potential of stromal cell cultures

The cultured stromal cells appeared morphologically as typical fibroblasts at all stages of culturing. The original primary cultures (passage 0) contained not only fibroblast-like cells, but also macrophages and individual epithelial cells or even their clusters (data not shown). However, after passage 2–3, the primary cultures appeared as morphologically homogeneous populations of fast-growing fibroblast-like cells. Proliferating cells in established cultures had a characteristic spindle-like shape with elongated projections. The cells became growth arrested at confluence. Stromal cells from various tissues had no significant differences in terms of cell morphology (Figure 1A–F). To determine the proliferative potential of the established stromal cell cultures, cells were grown with a subculturing ratio of 1 : 4 for a period of several months, until they have reached their proliferative senescence. The maximum number of passages varied from 7 to 25, which corresponds to approximately 14–50 cell divisions, as observed from Table 2. The average number of cell doublings for normal stromal lung cells was 32.6±12.6, whereas for tumour stromal lung cells it was somewhat higher (39.2±7.6). Remarkably, in all the analysed pairs of normal *vs* tumour stromal cell cultures, the proliferation capacity of the tumour stroma was higher than that of matched normal cell culture (Table 2, *P*=0.003, paired *t*-test). To estimate cell proliferation rates of tumour and normal stromal cultures, we used staining with Hoechst 33258 to plot cell growth curves at passage 6 of culturing. Except only one case (patient 13, metastatic chondrosarcoma in lung), the proliferation rate of tumour stromal cells exceeded that of normal cells. The cells of L2T and L2N stromal cultures proliferated with similar rates (Supplementary Figure 1a).

We also performed immunofluorescent staining of tumour and normal cell cultures (L2T, L2N, L6T, L6N, L7T, L7T, L15T, E1N, E1T, P1P, P3T, P4T) with antibodies to the nuclear proliferation antigen Ki67 (Supplementary Figure 1b). The relative content of Ki67-positive cells in the analysed cultures varied between 28 and 78%. The Ki67 labeling index for tumour stromal cells was statistically significantly higher than that for matched normal stromal cells (Supplementary Figure 1c).

### Mutational analysis of *TP53* and *KRAS2*

To determine the genetic status of cells in the obtained stromal cultures, we isolated genomic DNA from L5N, L5T, L6N, L6T, L13N, L13T, L15T, E1N, E1T, P1P, P3T and P4T cells, and then analysed the *TP53* gene (exons 5–7) by direct DNA sequencing and the *KRAS2* gene (codon 12) using highly sensitive mutant-allele-specific amplification. We did not detect any mutations in the *TP53* gene of tumour stromal cells (L5T, L6T, L13T, L15T, E1T, P3T, P4T), as well as of normal stromal cells (L5N, L6N, L13N, E1N, P1P). Similarly, no activating mutations were revealed in codon 12 of the *KRAS2* gene (Supplementary Figure 2) in both tumour and normal stroma cells. These results suggest that stromal cells of the tumours studied are generally genetically normal.

### Detection of epithelial-like cells in stromal cell cultures

**Figure 2:**
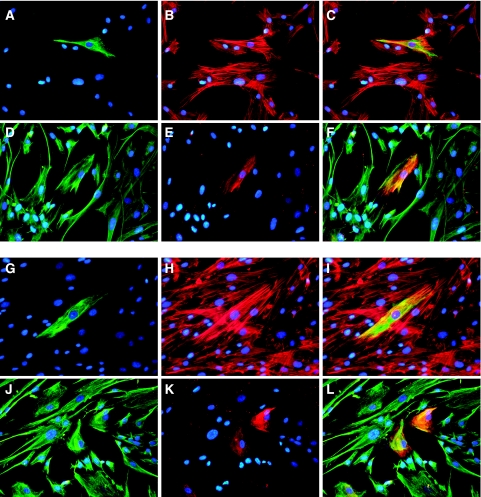
Indirect immunofluorescence staining of tumour (L2T, **A**–**F**) and normal (L2N, **G**–**L**) stromal cells. The L2T tumour stromal cells stained with mouse anti-pancytokeratin antibody (**A**), phalloidin –TRITC (**B**), mouse anti-vimentin antibody (**D**) and with rabbit anti-pancytokeratin antibody (**E**). (**C**) merge of **A** and **B**; (**F**) merge of **D** and **E**. The L2N normal stromal cells stained with mouse anti-pancytokeratin antibody (**G**), phalloidin –TRITC (**H**), mouse anti-vimentin antibody (**J**) and with rabbit anti-pancytokeratin antibody (**K**). (**I**) merge of **G** and **H**; (**L**) merge of **J** and **K**. All slides were counterstained with DAPI. Original magnification × 200.

To define cell types within the prepared stromal cultures, we analysed the expression of vimentin as a common mesenchymal cytoskeletal marker. An immunofluorescence analysis performed using monoclonal antibodies V9 on cells of normal (L1N, L2N, L5N, L6N, L7N, L8N, L13N) and tumour stromal cultures (L1T, L2T, L5T, L6T, L7T, L8T, L13T, L16T, P3T) revealed that practically all these cultures were positive for expression of intracellular vimentin suggesting that they belonged to a mesenchymal cell type (Table 2 and Figure 2D and J).

We used monoclonal anti-pancytokeratin antibodies C-11 to analyse cells expressing cytokeratins, markers of cells of epithelial origin. Surprisingly, we detected cytokeratin-positive cells in a number of cultures of normal (L2N and L5N) and tumour stromal cells (L2T, L5T, L7T, L16T). The total content of cytokeratin-positive cells in other analysed cell cultures (L1N, L1T, L6N, L6T, L7N, L13N, L13T and P3T) was rather low – <0.1% of the total number of cells (Table 2). The detected cytokeratin-positive cells had an elongated morphological appearance characteristic of fibroblasts (Figure 2). To further characterise the cytokeratin-positive cells, we used double immunofluorescent staining with rabbit anti-pancytokeratin antibodies and monoclonal anti-vimentin antibodies V9. Figure 2 shows the result of this staining in L2N and L2T cell cultures, clearly evidencing that cells expressing cytokeratin are also vimentin-positive. Similar results were obtained for stromal cell cultures L5N, L5T, L7T and L16T (data not shown).

### Protein expression patterns of stromal cells

**Figure 3:**
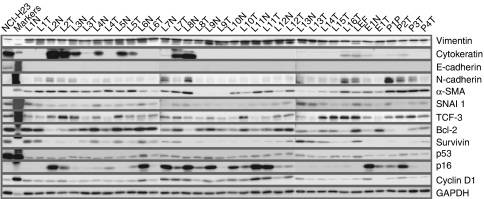
Western blot analysis of vimentin, cytokeratins, E-cadherin, N-cadherin, *α*-SMA, SNAI I (Snail) protein, TCF3 (E2A), Bcl-2, survivin, p53, p16 and cyclin D1 expression in tumour and matched normal stromal cells. Mouse monoclonal anti-GAPDH antibody was used as a loading control. NCI-H23 – the human lung adenocarcinoma NCI-H23 cell line; M – protein molecular weight marker (Santa Cruz).

We performed western blot analysis on cellular extracts from cells obtained at early stages of culturing (passages 4–6). All tumour and normal stromal cells expressed the mesenchymal protein marker vimentin (Figure 3). The protein level of vimentin expression was approximately the same for all cell culture extracts, consistent with the results of a previously performed immunostaining. Western blot analysis of cellular extracts with monoclonal antibodies C-11 allowed us to estimate the expression of cytokeratins in cells of stromal cultures, including some of those not studied by immunofluorescence (Figure 3). In a number of normal (L2N, L3N, L4N, L5N, L7N and L8N) and tumour (L2T, L3T, L5T, L7T and L16T) cell cultures, expression of cytokeratins was detected, suggesting the presence of cytokeratin-positive cells. In the case of oesophagus tumour, expression of cytokeratins was detected in cells of the normal stromal culture and was relatively low in the tumour stroma. In pancreatic stromal cultures, expression of cytokeratins was detected in cells prepared from a patient with chronic pancreatitis and a patient with carcinoid of pancreas. In two cases with ductal adenocarcinoma of pancreas, stromal cells were found to be cytokeratin-negative. It is worth noting that cytokeratin expression was detected in two cell cultures (L7N and P2T) that seemed cytokeratin-negative from fluorescent immunostaining.

The other marker of epithelial cells, E-cadherin, was not expressed in all stromal cultures analysed. Interestingly, at the same time practically all cultures showed low or moderate expression of N-cadherin (Figure 3).

To check for possible presence of myofibroblast cells in the prepared stromal cultures, we used a western blot analysis of cell extracts with monoclonal antibody 1A4 against *α* -smooth muscle actin (*α*-SMA) – a protein marker of myogenic fibroblast differentiation (Figure 3). We failed to observe the expected enhanced expression of *α*-SMA in tumour stromal cell cultures: *α*-SMA was expressed in both tumour and normal stromal cells. Moreover, in some cases (L6N, L8N, L10N, L11N and E1N) the expression of *α*-SMA was even higher in normal stromal cells than in the corresponding tumour stromal cells. The highest level of *α*-SMA was detected in the stromal culture prepared from the pancreatitis tissue specimen and in all tumour stromal cultures of pancreas cancer. This observation is consistent with the data on frequent development of tumour desmoplasia in this organ.

Finally, we analysed the expression patterns of seven important cellular oncoproteins (survivin, p53, cyclin D1, p16, BCL-2, SNAI I and TCF-3) whose expression levels in various human tumours are frequently different from the levels in normal cells (Figure 3). In tumour stromal cell cultures L1T, L4T, L5T, L6T, L9T and L13T, the level of survivin was lower than in the corresponding normal stroma cell cultures. Interestingly, in some of the normal cell cultures the expression level of survivin was close to that in cells of lung adenocarcinoma NCI-H23. In other cases, the level of survivin expression was rather low, although slightly increased in a number of tumour stroma cell cultures (L2T, L3T, L7T, L8T, L11T and L12T) as compared with normal stromal cells. We did not reveal any significant correlation between the expression levels of survivin and cytokeratins in the analysed cell cultures. In several tumour stromal cell cultures (L1T, L5T and L7T), the expression level of the tumour suppressor p53 protein was slightly higher than in normal stromal cells, whereas in other cultures (L6T, L9T and L13T) it was, in contrast, somewhat lower. Nevertheless, the level of p53 in both normal and tumour stromal cells was rather low suggesting the absence of p53 mutations in the cell cultures studied. Similarly, the level of cyclin D1 protein in tumour stromal cell cultures was higher (L2T, L3T, L5T, L7T and L12T) or lower (L6T, L8T, L9T, L10T and L13T) than in the corresponding normal stromal cell cultures. Interestingly, in the case of stromal cells derived from a surgical tissue specimen of squamous cell cancer of oesophagus, the cyclin D1 expression in tumour stromal cells was downregulated as compared with that observed in normal stromal cells. The western blot analysis showed that the expression level of p16 in tumour stromal cells had often been lower than in normal stromal cultures (Figure 3). The expression level of the antiapoptotic protein Bcl-2 in the stromal cultures obtained was heterogeneous: in some tumour stromal cultures (L2T, L3T, L7T, L8T, L10T, L11T and E1T) the Bcl-2 expression was upregulated, whereas in some others (L1T, L4T, L5T, L9T and L13T) downregulated. Interestingly, stromal cells of all tumour and normal cultures expressed detectable quantities of the SNAI I (Snail) and TCF-3 (E2A) proteins known to be transcriptional repressors of the *CDH1* gene and potential inducers of EMT.

Finally, we estimated the expression levels of the proteins under study in 16 cultures of lung tumour stromal cells and in 13 cultures of normal lung stromal cells (Supplementary Figure 3). It was shown that of 12 proteins studied only p16 was statistically significantly (*P*=0.02, two-tailed unpaired *t*-test) downregulated in lung tumour stroma as compared with normal lung stroma.

## Discussion

Cancer cell lines derived from human tumours are an extremely important research tool for understanding basic tumour biology and tumour intervention strategies. It was shown that established human cancer cell lines retain morphological, phenotypic and genetic characteristics of their corresponding parental tumours (Wistuba *et al*, 1998, 1999). We assumed that stromal cell cultures established from human tumours might be representative of the corresponding tumour microenvironment thus providing a useful complement to cancer cell lines in human neoplasia research. In this study, we describe preparation of a panel of stromal cell cultures derived from several types of human tumours and from normal cancer-free tissue. A majority of these cultures were derived from surgically resected primary tumours of lung, but the panel includes also stromal cell cultures prepared from oesophagus and pancreas cancer tissues. Stromal cells from tumours of different tissues had no significant differences in terms of cell morphology. Microscopically, all established stromal cultures after 3–4 passages grew as a monolayer of cells with fibroblast-like appearance, devoid of normal or malignant epithelial cells. Several lines of evidence suggest that stromal cell senescence can greatly contribute to age-related pathologies including cancer (Parrinello *et al*, 2005). But in all our experiments, stromal cells derived from normal part of the resected lung tumour tended to senesce faster (after fewer population doublings) than cells from tumour part. Along with increased replicative lifespan, tumour stromal cells were characterised by higher values of the Ki67 index relative to normal stromal cultures. The cell proliferation curves also suggest that tumour stromal cells proliferate more rapidly than normal cells, at least at culture passages 4–6. All these data show that the *in vitro* proliferative potential of tumour-associated fibroblasts is higher than that of their matched normal cells. It is assumed that at least in some cases the intratumoural fibroblast population has a higher replicative capacity than surrounding peritumoural stromal cells.

Epithelial to mesenchymal transition is a fundamental biological process in which epithelial cells lose their polarity and adopt fibroblast-like morphology appropriate for migration (Thiery, 2002; Grunert *et al*, 2003). In recent papers, active EMT processes were described for lung epithelial cells under different pathological conditions such as lung fibrosis and cancer (Iwano *et al*, 2002; Kim *et al*, 2006). We also found possible signs of an EMT process in the established lung tumour and matched normal stromal cell cultures. In particular, generally quite homogeneous mesenchymal vimentin-positive cells in all examined cultures contained a portion of cytokeratin-positive cells. These cells persisted in populations of both tumour and normal stromal cells (Figure 3). The double-positive cells might well emerge as a result of an EMT process in tumours and surrounding tissues. Cytokeratin-positive cells with a fibroblast-like phenotype in the tissue-surrounding dissected tumours may also be a sign of a high incidence of field cancerisation in the upper aerodigestive tract of lung cancer patients. The source of these cytokeratin-positive fibroblast-like cells remains uncertain. They can originate from transdifferentiated normal pulmonary epithelial cells as described for pathological pulmonary fibrogenesis (Iwano *et al*, 2002; Kim *et al*, 2006). The lack of mutations in the *TP53* and *KRAS2* genes in a number of tumour stromal cultures analysed supports this hypothesis. The tumour cells can also undergo EMT during their progression toward the final metastatic phenotype (Shintani *et al*, 2008). Both possibilities require additional experiments that are currently under way.

Moreover, one cannot exclude the possibility that stromal cells in the cultures obtained may undergo the reverse process, mesenchymal-to-epithelial transition (MET), because of which epithelial cells with acquired mesenchymal properties can recapitulate the initial epithelial state (Wells *et al*, 2008). Detectable quantities of the SNAI I (Snail) and TCF-3 (E2A) proteins in tumour and normal stromal cultures may also indicate active EMT/MET processes both in tumour stroma and surrounding presumably normal tissue.

To better characterise the prepared stromal cell cultures, we used western blot analysis to determine the expression profiles of some proteins involved in tumour cell differentiation and in different stages of malignant transformation and tumour progression. We observed practically homogeneous expression of vimentin in all stromal cultures obtained. These cultures were also E-cadherin negative, which suggests the lack of contamination with epithelial tissue. An expression of *α*-SMA that reflects activation and myogenic differentiation of stromal fibroblasts (Oda *et al*, 1990; Rønnov-Jessen *et al*, 1995; Rønnov-Jessen and Petersen, 1996) was also detected in most of tumour and normal stromal cultures and was the highest in the pancreas-derived stromal cells. Particularly interesting in our experiments was the observation of a very heterogeneous expression pattern of cytokeratin proteins in tumour and normal stromal cells. This finding may reflect active EMT in tumour and normal tissue in the lung of cancer patients. Moreover, the N-cadherin expression revealed in tumour and normal stromal cells may suggest an E-cadherin/N-cadherin switch, which is often a characteristic of EMT (Hazan *et al*, 2004). In addition, the detection of survivin expression in prepared stromal cell cultures from lung tumours and from matched normal lung tissue was also an unexpected result. In some stromal cell cultures, the survivin level may be similar to that in the established tumourigenic lung adenocarcinoma NCI-H23 cell line. Survivin expression is usually undetectable in most normal adult tissues, but survivin was identified as a tumour-associated protein highly expressed in a wide range of human carcinoma cells including lung (Monzo *et al*, 1999; Oshita *et al*, 2004). Several mechanisms have been suggested to account for this ‘overexpression’, one of them being the loss of the p53 tumour suppressor protein that inhibits transcription of the survivin gene in normal cells (Xia and Altieri, 2006; Altieri, 2008). Recently, inactivating mutations in the p53 tumour suppressor gene have been found in cancer stromal cells of prostate and breast tumours (Hill *et al*, 2005; Kiaris *et al*, 2005). However, we did not find here mutations in *TP53* or activating mutations in the *KRAS2* gene of stromal cells from lung, oesophagus and pancreas tumours. We showed also that the level of the p53 protein in all but one analysed stromal cultures was relatively low suggesting the wild-type status of p53 in most cultures (Carbone *et al*, 1994; Toyooka *et al*, 2003), and that the survivin level did not correlate with the level of p53. Therefore, inactivation of the p53 function can hardly explain the survivin level enhancement. An interesting suggestion may be that survivin expression is being changed in the course of the above mentioned EMT, considering that EMT induction leads in some cases to the emergence of cells with the phenotype of stem cells (Mani *et al*, 2008). Thus, high level of survivin may be a signature of some epigenetic or genetic changes occurring in host cells and inherited by their cell descendants. This interesting effect needs further investigation.

Summarising, a panel of stromal cell cultures from different human carcinomas and from matched normal cancer-free tissue was established. Using proliferating cell cultures of this panel, we showed that tumour stromal cells had an extended replicative lifespan and a proliferative potential in culture compared with matched normal stromal cells. Double immunofluorescence and western blot analysis of differentiation markers revealed a fraction of vimentin/cytokeratin double-positive cells in some established tumour and normal cultures. Thus, this study provides a new evidence of the EMT or MET processes in tumour and normal lung tissue of cancer patients. A western blot analysis of selected known oncoproteins revealed a complex pattern of their expression with a common feature – a low level of the p53 protein, indicating that it is the wild-type protein. Unexpectedly, quite a proportion of stromal cells were shown to express high level of survivin, which is characteristic of embryonic and tumour cells. Established primary cultures can serve as a valuable source for isolation and characterisation of different stromal cell populations.

## Figures and Tables

**Table 1:** Characteristics of patients and patient tumours

**Patient #**	**Sex**	**Age**	**Histological classification**	**TNM**	**Stromal cell cultures**
1	F	64	AD of lung	T1N1M0	L1T, L1N
2	M	57	AD of lung	T3N2M1	L2T, L2N
3	M	56	AD of lung	T4N2M0	L3T, L3N
4	M	55	SCC of lung	T4N0M0	L4T, L4N
5	F	41	SCC of lung	T2N1M0	L5T, L5N
6	M	58	SCC of lung	T4N0M0	L6T, L6N
7	M	56	SCC of lung	T3N2M0	L7T, L7N
8	M	50	SCC of lung	T2N0M0	L8T, L8N
9	M	66	AD of lung	T1N2M0	L9T, L9N
10	M	54	SCC of lung	T2N2M0	L10T, L10N
11	M	56	AD of lung	T3N0M0	L11T, L11N
12	F	53	BAC of lung	T2N0M0	L12T, L12N
13	M	16	Metastatic chondrosarcoma	n/a	L13T, L13N
14	M	59	AD of lung	T1N0M0	L14T
15	M	66	SCC of lung	T2N2M0	L15T
16	M	69	SCC of lung	T3N0M0	L16T
17	F	42	SCC of oesophagus	T2N2M0	E1T, E1N
18	F	34	CA of pancreas	n/a	P2T
19	M	62	AD of pancreas	T3N0M0	P3T
20	M	51	AD of pancreas	T3N1aM0	P4T

Abbreviations: AD=adenocarcinoma; BAC=bronchioloalveolar carcinoma; CA=carcinoid; n/a=data not available; SCC=squamous cell carcinoma.

**Table 2:** Characteristics of stromal cell cultures

**Stromal cell cultures**		**Replicative lifespan of cells *in vitro*^a^**		**Vimentin-positive cells in cultures^b^**		**Cytokeratin-positive cells in cultures^b^**	
**Tumour**	**Normal**	**Tumour**	**Normal**	**Tumour**	**Normal**	**Tumour**	**Normal**
L1T	L1N	20 (40)	18 (36)	100%	100%	>0.1%	>0.1%
L2T	L2N	21 (42)	19 (38)	100%	100%	5.7%	5.3%
L3T	L3N	19 (38)	14 (28)	ND	ND	ND	ND
L4T	L4N	25 (50)	24 (48)	ND	ND	ND	ND
L5T	L5N	20 (40)	18 (36)	100%	100%	1.6%	2.7%
L6T	L6N	18 (36)	14 (28)	100%	100%	>0.1%	>0.1%
L7T	L7N	15 (30)	8 (16)	100%	100%	2.9%	>0.1%
L8T	L8N	18 (36)	7 (14)	100%	100%	ND	ND
L10T	L10N	17 (34)	14 (28)	ND	ND	ND	ND
L13T	L13N	29 (58)	27 (54)	100%	100%	>0.1%	>0.1%
L14T	n/a	17 (34)	n/a	ND	ND	ND	ND
L15T	n/a	20 (40)	n/a	ND	ND	ND	ND
L16T	n/a	16 (32)	n/a	100%	n/a	2.2%	n/a
E1N	E1T	21 (42)	11 (22)	ND	ND	ND	ND
P3T	n/a	13 (26)	n/a	100%	n/a	>0.1%	n/a

Abbreviations: n/a=data not available; ND=not done.

a Limit of passages (maximal number of cell doublings) *in vitro* with subcultivation ratio of 1 : 4.

b Percentage of total cells.
