# Supplementary material for: Cellular and molecular phenotypes of proliferating stromal cells from human carcinomas
Source: Br J Cancer. 2010 Apr 20;102(10):1533–40. doi: 10.1038/sj.bjc.6605652 (PMC2869161; doi:10.1038/sj.bjc.6605652)
Supplement: Supplementary Figure 1 [file 6605652x1.doc]

**Supplementary Figure 1**

**A**

| **Lung** | | |
| --- | --- | --- |
| Culture | Ki67 Index | *P* value |
| L2N | 65.6 ± 7.4 | *P<0.05* |
| L2T | 78.1 ± 7.2 |
| L6N | 30.8 ± 6.5 | *P<0.005* |
| L6T | 71.9 ± 8.8 |
| L7N | 22.5 ± 5.8 | *P<0.005* |
| L7T | 63.5 ± 11.3 |
| L15T | 75.5 ± 8.7 | N/A |
| **Esophagus** | | |
| Culture | Ki67 Index | Probability |
| E1N | 28.5 ± 5.6 | *P<0.05* |
| E1T | 39.6 ± 7.4 |

| **Pancreas** | | |
| --- | --- | --- |
| Culture | Ki67 Index | Probability |
| P1P | 46.7 ± 8.1 | N/A |
| P3T | 55.9 ± 6.9 | N/A |
| P4T | 66.7 ± 6.7 | N/A |

**B**

**C**

**E1N**


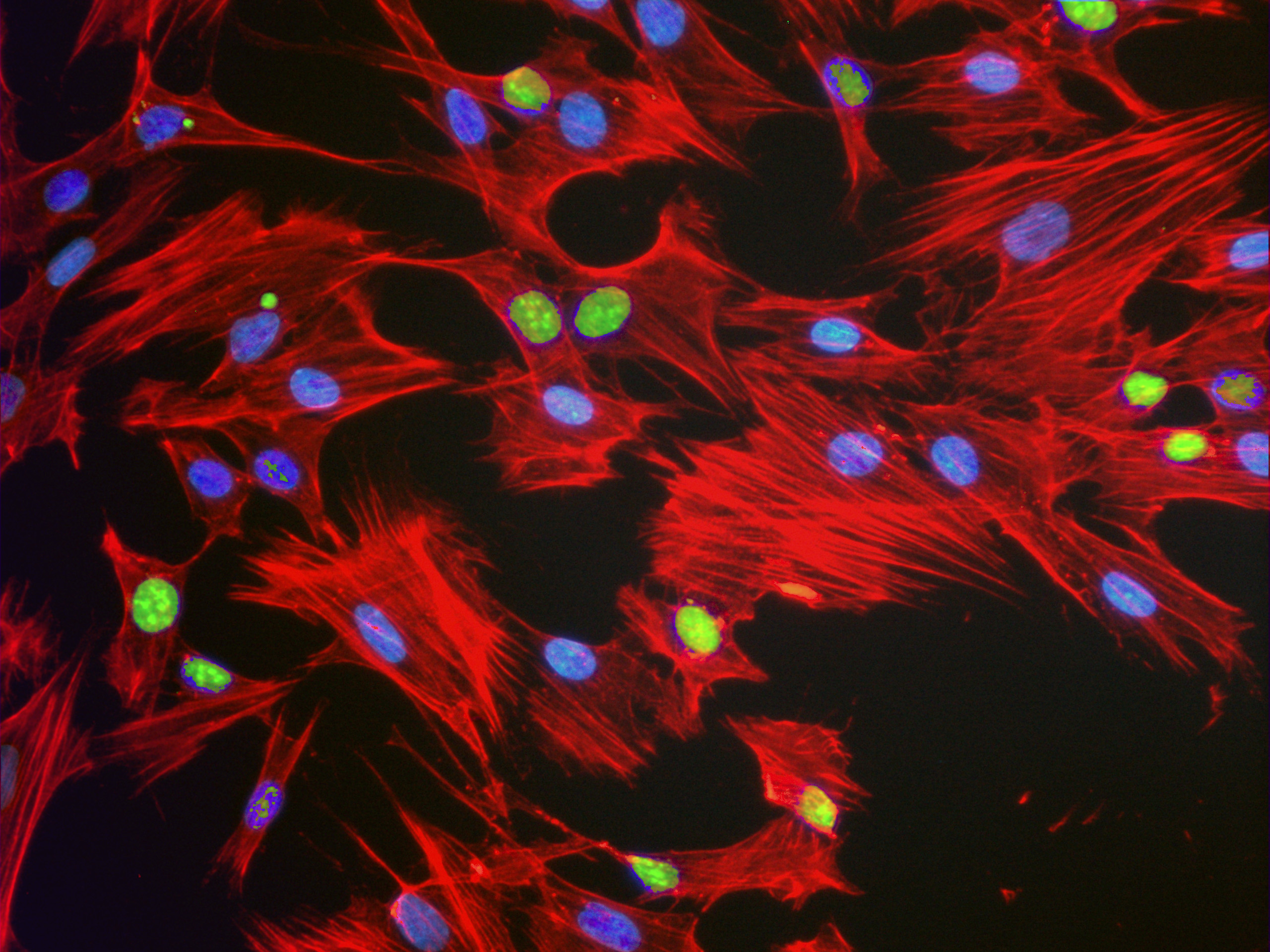


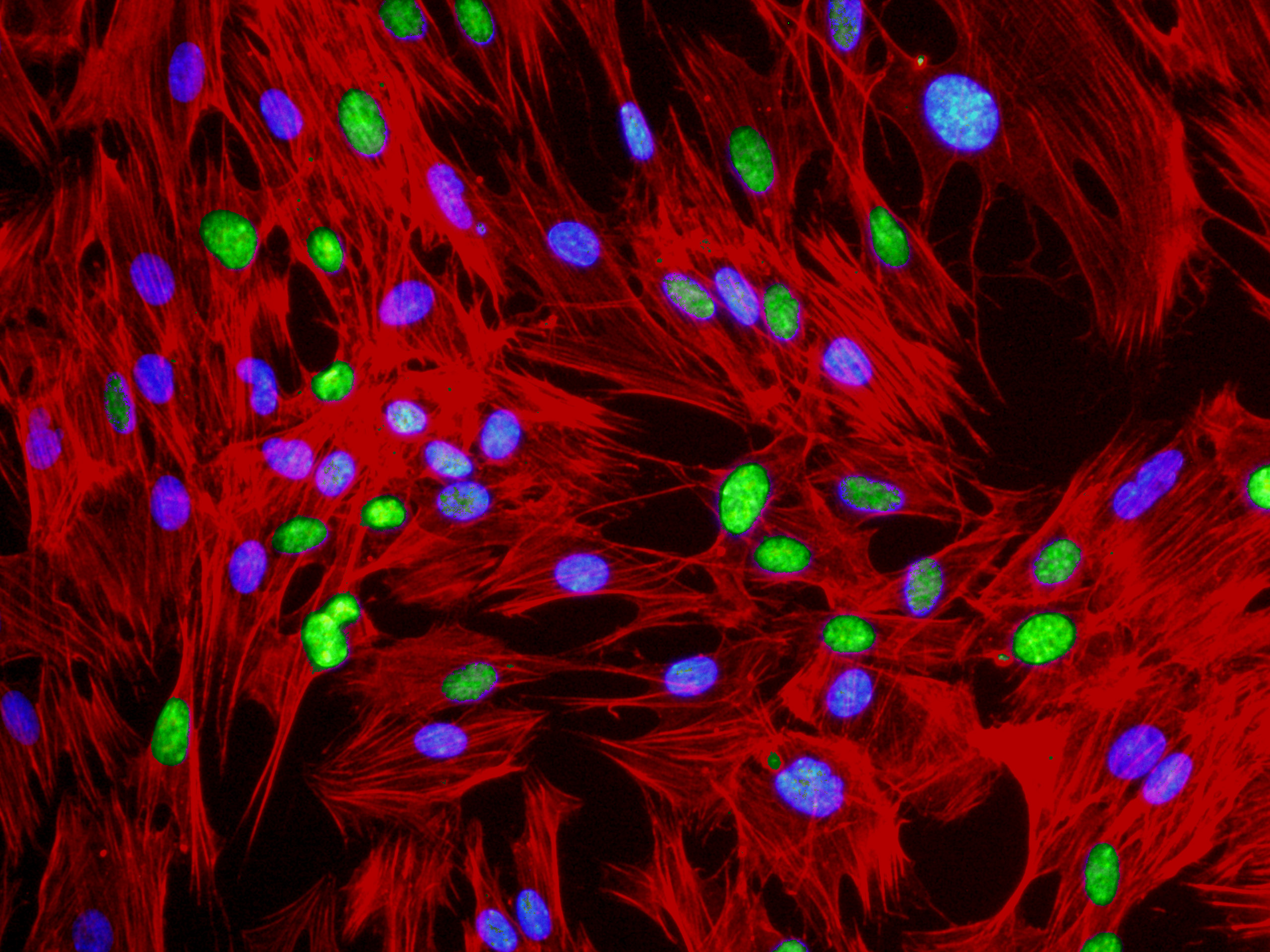


**E1T**
