# Supplementary figures and images for: Cellular and molecular phenotypes of proliferating stromal cells from human carcinomas
Source: Br J Cancer. 2010 Apr 20;102(10):1533–40. doi: 10.1038/sj.bjc.6605652 (PMC2869161; doi:10.1038/sj.bjc.6605652)

**Supplementary Figure 2**


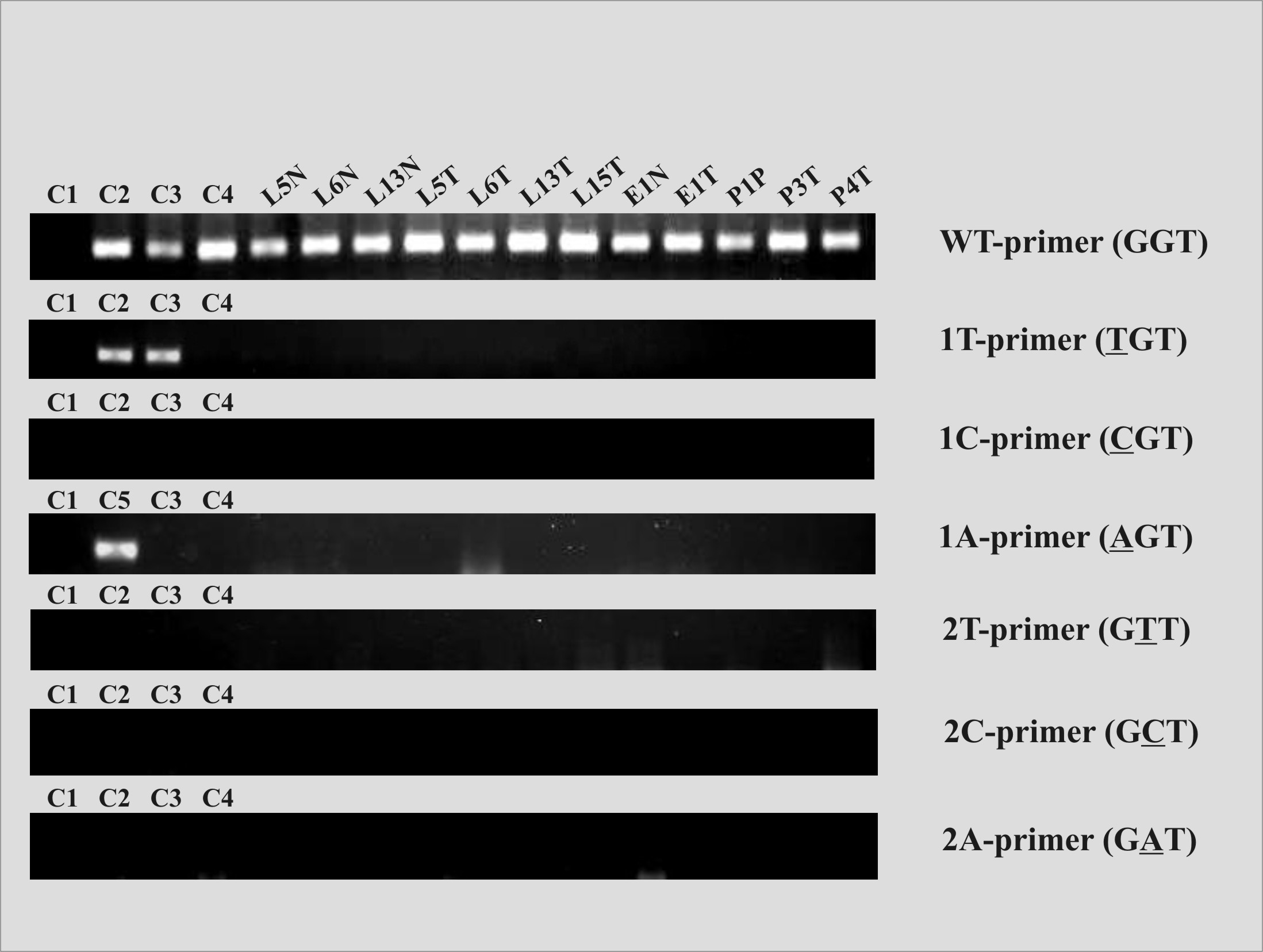

Supplement: Supplementary Figure 2 [file 6605652x2.doc]
