# Supplementary material for: Cellular and molecular phenotypes of proliferating stromal cells from human carcinomas
Source: Br J Cancer. 2010 Apr 20;102(10):1533–40. doi: 10.1038/sj.bjc.6605652 (PMC2869161; doi:10.1038/sj.bjc.6605652)
Supplement: Supplementary Figure 3 [file 6605652x3.doc]

**Supplementary Figure 3**
