# Supplementary material for: Cellular and molecular phenotypes of proliferating stromal cells from human carcinomas
Source: Br J Cancer. 2010 Apr 20;102(10):1533–40. doi: 10.1038/sj.bjc.6605652 (PMC2869161; doi:10.1038/sj.bjc.6605652)
Supplement: Supplementary Figures legends [file 6605652x4.doc]

**Supplementary figure legends:**

**Supplementary figure 1**

Growth characteristics of the cultivated tumor and normal stromal cells. (**A**) Growth curves for tumor and normal stromal cultures. RFU, relative fluorescence units. (**B**) Indirect immunofluorescence staining of normal (E1N) and tumor (E1T) stromal cells with anti-Ki67 antibody and phalloidin–TRITC. Slides were counterstained with DAPI. Original magnification ×200. (**C**) Mean values of Ki67 scores in normal and tumor stromal cells. The Ki67 index was defined as a percentage of Ki67 positive nuclei. Data are presented as the mean ± SEM. *P‑*values were determined by a two-tailed unpaired Student’s *t*-test.

**Supplementary figure 2**

Mutant‑allele-specific PCR amplification analysis of mutations at codon 12 of the *KRAS2* gene. As positive controls for codon 12 mutation of the *KRAS2* gene, genomic DNA of NCI-H23 (C2), A549 (C5) and MIA PaCa-2 (C3) cancer cell lines were used. As negative controls, water (C1) and genomic DNA of normal lung embryonic fibroblasts (C4) were used.

**Supplementary figure 3**

Densitometric quantitation of protein expression levels in 13 normal lung (cell cultures L1N-L13N, Table 1) and in 16 lung tumor (cell cultures L1N-L16N, Table 1) stromal cultures. The digital images of Western blot bands were quantified by densitometric scanning using the Bio-Rad Quantity One software program, and the expression levels were normalized to the GAPDH expression level. Data are presented as the mean ± SEM. *P*-values were determined by a two‑tailed unpaired Student’s *t*-test.
