# Supplementary material for: Cellular and molecular phenotypes of proliferating stromal cells from human carcinomas
Source: Br J Cancer. 2010 Apr 20;102(10):1533–40. doi: 10.1038/sj.bjc.6605652 (PMC2869161; doi:10.1038/sj.bjc.6605652)
Supplement: Supplementary Table 1 [file 6605652x5.doc]

**Supplementary Table1** List of the primers used for PCR amplification of genomic DNA

| **Primer designation** | **Sequence (5’- 3’)** |
| --- | --- |
| ***p53-5Exon reverse primer*** | **GCC CCA GCT GCT CAC CAT CGC TA** |
| ***p53-5Exon forward primer*** | **CTC TTC CTA CAG TAC TCC CCT GC** |
| ***p53-6Exon reverse primer*** | **GGC CAC TGA CAA CCA CCC TTA ACC** |
| ***p53-6Exon forward primer*** | **GAT TGC TCT TAG GTC TGG CCC CTC** |
| ***p53-7Exon reverse primer*** | **CAA GTG GCT CCT GAC CTG GAG TC** |
| ***p53-7Exon forward primer*** | **GTG TTA TCT CCT AGG TTG GCT CTG** |
| ***KRAS-WT reverse primer*** | **CTC ATG AAA ATG GTC AGA GAA ACC** |
| ***KRAS-WT forward primer*** | **TGT GGT AGT TGG AGC TGG T** |
| ***KRAS-1T primer*** | **ACT TGT GGT AGT TGG AGC TT** |
| ***KRAS-1C primer*** | **ACT TGT GGT AGT TGG AGC TC** |
| ***KRAS-1A primer*** | **ACT TGT GGT AGT TGG AGC TA** |
| ***KRAS-2T primer*** | **CTT GTG GTA GTT GGA GCT GT** |
| ***KRAS-2C primer*** | **CTT GTG GTA GTT GGA GCT GC** |
| ***KRAS-2A primer*** | **CTT GTG GTA GTT GGA GCT GA** |
